# International society of sports nutrition position stand: tactical athlete nutrition

**DOI:** 10.1080/15502783.2022.2086017

**Published:** 2022-06-23

**Authors:** Drew E. Gonzalez, Matthew J. McAllister, Hunter S. Waldman, Arny A. Ferrando, Jill Joyce, Nicholas D. Barringer, J. Jay Dawes, Adam J. Kieffer, Travis Harvey, Chad M. Kerksick, Jeffrey R. Stout, Tim N. Ziegenfuss, Annette Zapp, Jamie L. Tartar, Jeffery L. Heileson, Trisha A. VanDusseldorp, Douglas S. Kalman, Bill I. Campbell, Jose Antonio, Richard B. Kreider

**Affiliations:** a Exercise & Sport Nutrition Laboratory, Human Clinical Research Facility, Department of Health & Kinesiology Texas A&M University, College Station, TX, USA; b Texas State University, Metabolic and Applied Physiology Laboratory, Department of Health & Human Performance, San Marcos, TX, USA; c University of North Alabama, Department of Kinesiology, Florence, AL, USA; d University of Arkansas for Medical Sciences, Department of Geriatrics, Little Rock, AR, USA; e Oklahoma State University, Department of Nutritional Sciences, Stillwater, OK, USA; f US. Army-Baylor Master’s Program in Nutrition, Department of Nutrition, San Antonio, TX, USA; g Oklahoma State University, Department of Kinesiology, Applied Health, and Recreation, Stillwater, OK, USA; h Brooke Army Medical Center, Department of Nutritional Medicine, San Antonio, TX, USA; i United States Special Operations Command, Preservation of the Force and Family, Tampa, FL, USA; j Lindenwood University, Exercise and Performance Nutrition Laboratory, College of Science, Technology, and Health, St. Charles, MO, USA; k University of Central Florida, Institute of Exercise Physiology and Rehabilitation Sciences, School of Kinesiology and Physical Therapy, Orlando, FL, USA; l The Center for Applied Health Sciences, Canfield, OH, USA; m Fire Rescue Wellness, Montgomery, IL, USA; n Nova Southeastern University, Department of Psychology and Neuroscience, Fort Lauderdale, FL, USA; o Baylor University, Department of Health, Human Performance, and Recreation, Waco, TX, USA; p Research & Development, Bonafide Health LLC, Harrison, NY, USA; q Dr. Kiran C Patel College of Osteopathic Medicine, Nova Southeastern University, Nutrition Department, Davie, FL, USA; r University of South Florida, Performance & Physique Enhancement Laboratory, Exercise Science Program, Tampa, FL, USA; s Fight Science Laboratory, Nova Southeastern University, Department of Health and Human Performance, Davie, FL, USA

**Keywords:** Tactical athletes, occupational athletes, nutrition, ergogenic aids, police, law enforcement, LEO, fire, readiness, military, first responders

## Abstract

This position stand aims to provide an evidence-based summary of the energy and nutritional demands of tactical athletes to promote optimal health and performance while keeping in mind the unique challenges faced due to work schedules, job demands, and austere environments. After a critical analysis of the literature, the following nutritional guidelines represent the position of the International Society of Sports Nutrition (ISSN).

**General Recommendations:**

Nutritional considerations should include the provision and timing of adequate calories, macronutrients, and fluid to meet daily needs as well as strategic nutritional supplementation to improve physical, cognitive, and occupational performance outcomes; reduce risk of injury, obesity, and cardiometabolic disease; reduce the potential for a fatal mistake; and promote occupational readiness.

**Military Recommendations:**

Energy demands should be met by utilizing the Military Dietary Reference Intakes (MDRIs) established and codified in Army Regulation 40-25. Although research is somewhat limited, military personnel may also benefit from caffeine, creatine monohydrate, essential amino acids, protein, omega-3-fatty acids, beta-alanine, and L-tyrosine supplementation, especially during high-stress conditions.

**First Responder Recommendations:**

Specific energy needs are unknown and may vary depending on occupation-specific tasks. It is likely the general caloric intake and macronutrient guidelines for recreational athletes or the Acceptable Macronutrient Distribution Ranges for the general healthy adult population may benefit first responders. Strategies such as implementing wellness policies, setting up supportive food environments, encouraging healthier food systems, and using community resources to offer evidence-based nutrition classes are inexpensive and potentially meaningful ways to improve physical activity and diet habits. The following provides a more detailed overview of the literature and recommendations for these populations.

## 1. Introduction

**Figure 1.**
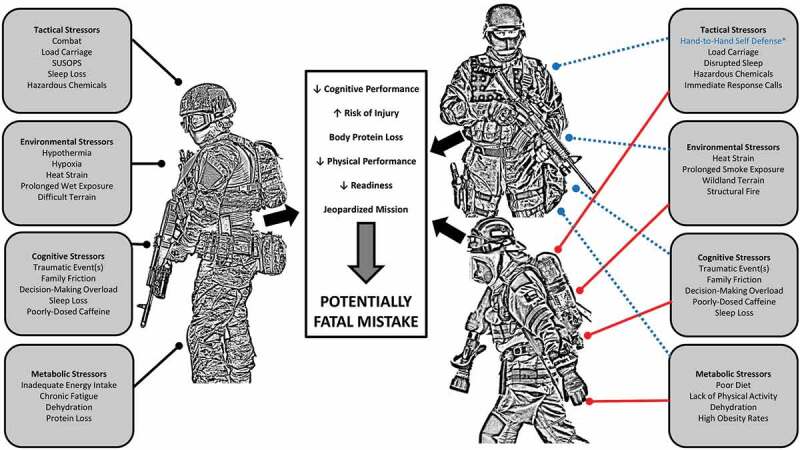
Tactical Athlete Stressors.

Military personnel, law enforcement officers, and firefighters perform some of our society’s most physically demanding jobs [1,2–4]. For instance, essential job tasks related to these occupations may include lifting and carrying heavy and odd objects and tools, casualty extraction, moving rapidly to obtain a tactical advantage or maintain safety, and forcible entry, as well as high cognitive demand, during life and death decisions under high stress and austere conditions. Typically, these tasks are performed while wearing personal protective equipment (PPE) weighing approximately 10–50 kg [5,6]. Based on the occupational demands and the need for performance-based training, these individuals are often referred to as ‘Tactical Athletes’ [7] (Figure 1).

In recent years, there has been an increased emphasis on developing evidence-based physical training methodologies and strategies aimed at maintaining and improving occupational and physical performance, as well as reducing the risk of injury and disease within these populations. Subsequently, the importance of providing nutritional guidance and identifying best practices for fueling tactical athletes has become paramount. This position stand aims to provide practical guidance and recommendations for tactical athletes with respect to evidence-based nutritional strategies that promote health and occupational performance.

## 2. Methods

International Society of Sports Nutrition (ISSN) position stands are invited papers the ISSN Editors and Research Committee identify as being of interest to scientists and practitioners. Editors and/or the Research Committee identify a lead author or team of authors to perform a comprehensive literature review. A review of the scientific literature was conducted related to the tactical athlete, tactical athlete nutrition, warfighters, first responders, supplementation, health, performance, and operational readiness. This was accomplished by conducting key word searches related to the tactical athlete, as well as nutrition, on each topic summarized using the National Institutes for Health National Library of Medicine PubMed.gov search engine. The draft is then sent to leading scholars for review and comment. The paper is then revised as a consensus statement and reviewed and approved by the Research Committee and Editors as the official position of the ISSN.

## 3. Part I – Military

### 3.1. Physiological and Energy Expenditure Demands of Military

#### 3.1.1. Duty-Specific Tasks and Common Stressors

The physiological demands placed upon military personnel vary considerably based on the physical requirements demanded of the operator (i.e. military occupation specialty [MOS]), the mission of the unit, and deployment status. Military populations are heterogeneous as some military personnel work on primarily sedentary jobs (i.e., financial management technician), while others may engage in near-continuous physical tasks (i.e., deploying special operations Operator). Regardless, it is important for the warfighter to be physically and mentally ready when deployments are initiated and the pace of daily operations increases (i.e., operational tempo [OPTEMPO]). The modern-day warfighter faces a barrage of occupational challenges such as load carriage, heavy lifting, offensive and defensive combat/maneuvers, sprinting, and casualty evacuations [8]. Given the varying or unknown challenges of multi-domain operations (and future warfare), it is important for military personnel to be both anaerobically and aerobically conditioned, with most military operations requiring steady-state effort and occupational-specific tasks for a protracted period of hours or days.

Prolonged operations may leave warfighters without adequate energy intake, which can lead to poor physical performance and decision-making [9]. Warfighters are commonly exposed to hypo-energetic states, particularly during sustained periods of training or combat operations, which may lead to large energy deficits (i.e., 2,500–4,500 kcal/day) [10,11]. The United States Army Research Institute of Environmental Medicine (USARIEM) conducted studies demonstrating decrements in muscular strength and power following severe weight loss among participants in the U.S. Army Ranger School [10,12]. These physiological stressors are typically coupled with sleep deprivation, which can further exacerbate decrements in physical and mental performance [13]. Indeed, Nindl et al. [12] reported the approximate caloric deficit to be 3,000 kcal/day during these periods of training and operations. This imbalance was largely responsible for the observed loss of body mass (~3.1%), poor sleep (~3.6 h over a 72-h duration), and self-reported increases in depression, anger, fatigue, tension, and confusion [12]. While unpredictable, warfighters should seek and exploit opportunities to maximize the delivery of nutrient-dense foods while continuing with regular physical training to maximize operational readiness. In general, implementing strategies to combat negative energy balance status will best prepare the warfighter during sustained periods of training and combat and other protracted periods of tactical-specific activities.

Military personnel are subjected to environmental stressors, such as heat, cold, and high altitude, which may impair their ability to perform occupational tasks. Unacclimated individuals may be required to travel to geographical locations on short notice with severe environmental conditions. While having ‘boots on the ground’ is important to establish a military presence in locations of conflict, warfighters might be subjected to limited time or opportunity to acclimatize to new environments. In this respect, inadequate heat adaptation can leave the warfighter more susceptible to heat-related illnesses such as exertional heat stroke [14,15]. Similar to other athletes, physically fit warfighters fare much better in terms of physiological adaptations and acclimatization [16,17], specifically those who are aerobically well trained [18]. In terms of high-altitude exposure (i.e., 1,500–3000 m, 3,000–5,000 m, and 5,000 m or above are considered high-, very high-, and extreme altitude levels), aerobic/submaximal activities can be compromised at higher altitudes due to decreases in barometric pressure. High-altitude cerebral edema, high altitudes pulmonary edema, and acute mountain sickness are all potential conditions that can impact the performance of military personnel conducting operations at high altitudes [19–21]. Common symptoms associated with acute mountain sickness include disorientation, headaches, nausea, vomiting, fatigue, dizziness, and irritability, which can all negatively affect physical and cognitive performance [22], while altitudes greater than 3,000 m have been demonstrated to impair psychomotor performance, reaction time, vigilance, memory, and logical reasoning [23,24]. Lastly, physical and cognitive performance decrements may also result from cold exposure (e.g., reductions in muscular strength) [20]. Military field operations conducted in cold environments may result in a reduction in physical performance (e.g., anaerobic power) [25]. Furthermore, the risk of dehydration in cold environments is a major concern [20]. It is important to note that these environmental stressors exacerbate these physiological responses in warfighters. These changes do not include the additional physical burden associated with load carriage requirements.

#### 3.1.2. Physical fitness considerations and energy expenditure

While Service members often engage in long work periods at submaximal intensities, various tasks may be high intensity in nature and include heavy load carriage; thus, it is also important that military personnel to possess adequate levels of muscular endurance, strength, and power. Traditionally, warfighters have been subjected to heavy load carriage, and that load has progressively increased over the past 10 to 20 years [26]. Loads include equipment (i.e., weapon systems, ammunition, and provisions) and PPE, such as ballistic helmets, body armor, load-bearing vests, chest rigs, patrol packs, and boots [27]. Energy cost [28,29], altered gait mechanics [4,30,31], and risk of injury [4,32,33] all increase with a concomitant decrease in neuromuscular function [34] as a result of the increased load carriage demands. The context in which the load is carried also plays an important part in energy cost. For example, the speed at which a warfighter is moving while carrying a heavy load may increase the energy cost of the task (depending on duration and terrain gradients) [29,35,36]. Moreover, warfighters with lower body mass might be more disadvantaged in carrying absolute loads due to increased cardiovascular strain and reduced neuromuscular function [34]. Given these occupational-specific demands, warfighters may be subject to high energy and neuromuscular function costs when engaged in tactical operations.

Studies have reported a range of energy requirements, as low as 9.8 MJ/day (2,342 kcals) among female combat support personnel [37] to as high as 29.8 MJ/day (7,122 kcals) for male Marines [38]. Special Operations Forces (SOF) have been reported to have much higher daily average energy requirements in comparison to the average support soldier (4,099 ± 740 kcals/day vs. 3,361 ± 939 kcal/day, respectively) [39]. As a result, it is likely that the daily energy expenditure of SOF likely exceeds the energy requirements as suggested by the military dietary reference intake (MDRI). Moreover, energy expenditures through specific training ranged from ~17,150 kJ/day (~4,100 kcals/day) [40] during Army Ranger School to ~21,750 kJ/day (~5,200 kcals/day) [41] during US Army Special Forces Assessment and Selection (SFAS) have been reported. Barringer et al. [42] identified high daily energy demands of various SOF depending on the types of training conducted. For example, Marine Corps Forces Special Operations Command (MARSOC), the Ranger Assessment and Selection Program (RASP), and US Army Special Forces Small Unit Tactics Training groups had a predicted daily energy expenditure of 6,317 kcal/day, 4,264 kcal/day, and 5,215 kcal/day, respectively [42]. Prediction of SOF energy requirements can aid dietetic personnel in planning an appropriate feeding regimen to meet operational demands and optimize warfighter health and performance [42].

#### 3.1.3. Duty-specific health and performance burdens

Much like the general population, warfighters are susceptible to various cardiometabolic diseases, such as heart disease [43]. Similar to first responders, warfighters are exposed to various physiological, psychological, and extreme environmental stressors that can exacerbate the development and progression of cardiometabolic disease, among other adverse health issues (i.e., exertional heat illness) [44]. Like greater segments of the general population, the prevalence of overweight and obese military personnel has increased [45], which is associated with various chronic conditions, such as cardiovascular disease, type 2 diabetes, and other inflammatory conditions, as well as the occurrence of a mental health challenge (i.e., major depressive disorder and anxiety). Although the military has a fitness standard in place to determine employability and occupational specialty, the US Army reported 17.3% of warfighters as being obese, in addition to 52.9% being classified as overweight [46]. This is problematic for multiple reasons, as warfighter readiness is likely compromised due to overweight and/or obesity classifications, and warfighters with obesity disproportionately use more healthcare services compared to those of normal weight [47], with musculoskeletal issues among the most common diagnostic codes identified. A recent cross-sectional study regarding the fiscal year 2015 military claims and treatment facility data noted obese warfighters utilized approximately 30% of the recorded healthcare services for musculoskeletal issues compared to non-obese warfighters [47]. Moreover, chronic sleep restriction can further exacerbate challenges associated with obesity among warfighters [48]. For example, chronic sleep restriction is associated with decreases in leptin and increases in ghrelin [49] as well as increased consumption of high-fat and high-carbohydrate foods [49–51]. Chronic sleep restriction also increases insulin resistance and impairs glucose tolerance [49,52–54]. In this respect, poor long-term sleep practices can lead to an increased risk of cardiovascular disease [55], musculoskeletal injuries [56], substance abuse [57], and post-traumatic stress disorder (PTSD) [58].

In terms of load carriage, heavy loads (31–44 kg) [59] routinely carried by warfighters consistently lead to injuries, most commonly to the lower back, which heavily impacts occupational performance, mission success, and readiness [60,61]. An association between fitness scores on the Army Physical Fitness Test (APFT) and lower back pain [62] has been established – specifically, a score below 200 may be predictive of lower back pain. On the contrary, higher fitness levels are likely protective against lower back pain and other musculoskeletal injuries [63]. While highly fit individuals are still susceptible to musculoskeletal injuries due to overuse, warfighters who are overweight or obese are at a greater risk of injury due to additional load (bodyweight) and physiological challenges [64,65]. Ultimately, strategies to prevent or reduce the risk of musculoskeletal injuries are important considerations for the warfighter, as injury will reduce occupational readiness and performance. Although there are physical fitness and body composition standards in place, military personnel are subjected to overuse and obesity-related issues that can reduce occupational readiness, physical performance, and overall health.

#### 3.1.4. Key points: physiological and energy expenditure demands of military

- The physiological demands military personnel face varies considerably depending on their intended mission; however, all warfighters should be prepared for high operational tempos and sustained training and combat conditions.
- The energy requirements of Special Operation Forces are not well understood. Greater research is needed to best prepare appropriate fueling regimens to meet the operational demands and optimize warfighter performance based on operational tasks and the environment in which they are working.
- Load carriage, chronic sleep loss, and environmental stressors impact warfighter health and performance and should be considered when developing fueling regimens to optimize mission success.

### 3.2. General energy and macronutrient guidelines for military personnel

Adequate nutrient intake can improve health as well as physical and occupational performance outcomes among warfighters [66]. Given that warfighters are often engaged in high-volume physical training, MDRIs have been established and codified in Army Regulation 40-25 to provide guidance on feeding for military personnel [67] and are based on moderate levels of activity suggested to be appropriate for personnel in garrison. In this regard, the recommended energy intake is 3,400 kcal/day for males and 2,300 kcal/day for females [67]. Based on Army Regulation 40-25, protein intakes for military personnel are suggested to be between 0.8 and 1.6 g/kg/day for both males and females and should be increased during periods of intense physical exertion and increased metabolic demand [67]. The reader is encouraged to review the ISSN position stands regarding protein intake for those engaged in intense training, as the recommendations are between 1.4 and 2.0 g/kg/day for exercising individuals [68,69]. The warfighter is likely to benefit from the ISSN’s recommended range, especially those who are engaged in regular exercise training. The current recommendations for carbohydrates and fats are based on well-established sports nutrition guidelines. In terms of carbohydrates, during prolonged periods of intense physical demand, it is recommended that warfighters consume between 4 and 8 g/kg/day [67]. Assuming adequate energy intake, warfighters who consume 50 to 55% of their total calories as carbohydrates will meet the recommended carbohydrate intake. Fat intake should be approximately 30% or less of total energy intake. Additionally, military personnel are advised to include the consumption of linoleic acid (17 g/day and 12 g/day for males and females, respectively) and alpha-linolenic acid (ALA: 1.6 g/day and 1.1 g/day for males and females, respectively) within their daily fat intake [67]. The MDRIs for energy intake and macronutrients are based upon height and weight measurements, representative of the 50^th^ percentile for military males and females and must be adjusted for body size, physical activity, environmental factors, clothing, equipment, and terrain, to be applicable to different Service members’ needs.

#### 3.2.1. Key points: general energy and macronutrient guidelines for military personnel

- The provision and timing of adequate amounts of energy, macronutrients, and micronutrients best promote optimal occupational performance outcomes and reduce injury and disease risk.
- Army Regulation 40-25 provides guidance on feeding for the warfighter.

### 3.3. Special nutritional considerations for military personnel

#### 3.3.1. Combating protein loss during energy deficit

Warfighters endure a combination of unique physiological, psychological, and environmental stressors during any period of sustained training and combat operations, which routinely result in a negative energy balance. As the magnitude of energy deficit increases, so does the rate of catabolism of whole-body and skeletal muscle protein [70]. Consuming adequate energy (via combat rations) to match energy expenditures and recommended protein intake [71,72] during ongoing training or combat would largely negate this protein catabolism. However, warfighters rarely achieve energy balance and optimal protein recommendations when subsisting on combat rations. During sustained training or combat, the operational tempo dictates what the warfighter normally eats and what is carried on their person. Energy deficits from increased energy demand can largely be attenuated with the provision and consumption of high calorie rations. While this dietary strategy is effective, it is an oversimplification of the well-recognized logistical and physiological barriers imposed upon the warfighter. These barriers include operational time constraints, appetite suppression, the inability to carry more food, or prioritizing operational items over rations. Thus, warfighters rarely achieve energy balance and optimal protein recommendations when subsisting on combat rations alone. This caloric deficit can lead to substantive losses in whole-body and skeletal muscle protein (i.e., negative protein balance) and, thus, profound performance decrements, especially with frequent and repeated exposures to energy deficits.

Depending on the length of the training or combat period, the extent of energy deficit can routinely achieve 40–70%. Negative protein balance has been largely unavoidable during these times and is further exacerbated by sub-optimal dietary protein intake (both quantity and quality) and the effects of limited energy intake [73,74]. Prolonged negative protein balance and concomitant muscle loss may compromise physical performance and increase injury risk and lost duty time, which further diminishes warfighter readiness [12,74]. While additional protein intake is effective in mitigating compromises in body protein homeostasis, especially when combined with resistance exercise [75], as the caloric deficit increases (~40% [73,75,76]) the anabolic effects of protein are absent, and the constituent essential amino acids (EAA) are prioritized as carbon skeletons for energy production.

Skeletal muscle sensitivity to anabolic capability is diminished in favor of whole-body protein homeostasis during caloric deficit [77,78]. For example, diet-induced energy deficits of 30% revealed that an EAA dose of 0.3 g/kg maintained whole-body protein balance and stimulated muscle protein synthesis, while a lower dose of 0.1 g/kg was only able to maintain protein balance [78]. A later study delivered 24 g of EAA after military-related tasks/exercises via three isonitrogenous formats (EAA-enriched whey protein formula, whey protein alone, or a mixed nutrient meal) and showed that muscle protein synthesis was not different between the groups; however, the EAA-enriched whey protein formula produced a significantly greater whole-body protein balance, followed by whey protein alone, and then the mixed nutrient meal [77]. Evidence encompassing a wide range of protein/EAA intake formats clearly indicates that the more easily digested formats can rapidly deliver EAA to the periphery for optimal stimulation of protein synthesis [79], while pragmatically this format may be better suited for a warfighter due to it being largely indestructible and resistant to spoilage, takes up little volume, and weighs very little. Taken together, these data strongly suggest that a supplemental format would be beneficial for the warfighter in the preservation of body protein homeostasis during extended periods of training, combat, and energy deficit.

#### 3.3.2. Key points: special considerations for military personnel

- Requirements of EAA increase with caloric deficit, such that supplementation should be considered.
- A greater peripheral delivery of EAA results in greater stimulation of both whole-body and muscle protein synthesis.
- Simpler EAA ingestion formats (i.e., free-form and protein alone) may provide for greater peripheral delivery of EAA and protein kinetic responses while also doubling as a more pragmatic consideration.

### 3.4. Hydration

#### 3.4.1. Considerations for hydration among military

Mission hydration recommendations from the Academy of Nutrition and Dietetics and the American College of Sports Medicine [80] suggest that the warfighters (1) consume 5 to 7 mL (~0.25 cups) per kilogram of body mass of water or sports drink 4 h prior to a mission, (2) limit dehydration (to <2% loss in body weight) during missions by consuming adequate amounts of fluids, and (3) consume 450 to 675 mL (2–3 cups) per 0.45 kg (1 lb) of body weight lost upon completion of the mission. Environmental demands may place additional fluid and electrolyte needs upon the warfighter and can compromise fluid homeostasis. Adequate fluid intake is critical in hot and humid environments to avoid heat-related injuries and early fatigue, which can hinder occupational performance while also posing a risk to overall health. Moreover, cold environments might reduce thirst [81]; thus, fluid intake may be reduced. This is even more problematic in high-altitude environments because hypoxia-induced increases in ventilation and diuresis can exacerbate fluid imbalance and likely are coupled with poor thirst sensation. The three-step approach to hydration, outlined by Cheuvront and Sawka [82] (see the Fist Responder Hydration section: Part 2), may be a helpful aid to the warfighter in minimizing the risk of dehydration.

### 3.5. Nutritional supplementation in warfighters

As highlighted throughout, daily training and operations of warfighters create physical and nutritional challenges that can compromise health, performance, and recovery. For these reasons, efficacious regimens of nutritional supplementation are commonly considered. As outlined in

Table 1, several nutritional supplements are commonly used by tactical athletes. In terms of promoting general health, the warfighter should consider ingesting a daily low-dose multivitamin (see Table 1) to ensure sufficient levels of vitamins and minerals are met in the diet [83]. While nutritional supplements are not intended to replace the role of whole foods within the tactical athlete’s diet, supplementation should be used as means to augment dietary intake with the purpose of achieving a health or performance benefit. Although most lack robust scientific support, beta-alanine, caffeine, and creatine monohydrate all have demonstrated merit in terms of safety, ergogenic potential, enhancing mental readiness, and neuroprotection.

**Table 1.** Commonly Used Supplements Consumed by Tactical Athletes.

Ingredient	Dosages	Function	Potential Benefit	Evidence to Support Efficacy and Safety
EAA	6–12 g [68,69]	Increase protein synthesis/turnover Increase recovery	Increase FFM and strength during training	Strong
Arginine	2–9 g (UL 20 g daily) [84]	Increase blood flow, nitric oxide	Increase exercise performance, increase growth hormone production, support immune function, and promote accretion of fat-free mass	Little to none
Citrulline	3–6 g [69]	Increase nitric oxide production	Increase aerobic and anaerobic performance	Mixed or limited
Beetroot juice or sodium nitrate	2–3 hours pre-exercise; 300–600 mg or 0.1 mmol/kg/d [69]	Increase blood flow	Improved work efficiently, reduced phosphocreatine degradation, improved time trial performance, and decreased blood pressure	Mixed or limited
Beta-alanine	4–6 g for 2–4 wks [87] or 4 x 0.8 to 1.6 g doses [69]	Increase carnosine, buffers acidity, and increase pH	Increase high intensity exercise capacity primarily in events lasting 30 sec to 4 min	Strong
HMB	1 g three times daily (3 g daily) [69]	Decrease protein breakdown, Increase protein synthesis	Promotes improvements in fat-free mass, decreases in fat mass, peak isometric force, and isokinetic torque production, improved aerobic performance, improved muscular strength, and attenuated muscle damage	Mixed or limited
BCAAs	6–14 g daily in 3:1:1 leucine to valine to isoleucine ratio [69]	Increase exercise capacity, Increase recovery	Improved psychological perception of fatigue (central fatigue), promote recovery, mitigate soreness, and loss of force production	Mixed or limited
Caffeine	3–6 mg/kg [97]	Increase energy, mood, endurance	Spare carbohydrate, improved endurance exercise capacity, improved alertness, improved cognitive function	Strong
Creatine Monohydrate	Load 4 x 5 g/d for 7- days; 3-5 g daily thereafter [69,116]	Increase energy/ATP, neuroprotection	Increase explosive sprint capacity, recovery from sprints, muscle endurance, and FFM gains during training	Strong
Omega-3 Fatty Acids	1–3 g/d	Regulation of blood pressure and vascular function	Cardio-protective benefits [85] and improved cognition	Strong
Glutamine	5 g or 0.3 g/kg [69]	Increase recovery, immune function	Tolerate stress, enhance strength, and muscle mass, improved glycogen stores, and improved recovery	Little to none
Melatonin	0.5–3 mg (starting with a lower dose is recommended) [84]	Regulate internal timing of biological rhythms to promote sleep	Improved sleep and optimize sleep quality [86]	Little to none
Multivitamin	Once daily	Supplements diet with vitamins and minerals in a state of deficiency	Improved vitamin status, support immune system*	Strong health benefits, limited ergogenic benefits*
Protein (e.g, Whey)	Variable, 20–40 g post-workout [68,69]**	Increase protein turnover/synthesis	Retention of lean body mass, positive effects on body composition, improved strength, reduced risk of injury	Strong
Glucosamine	1500 mg per day spread out over 3 doses	Osteoarthritis pain reduction	Slow cartilage degeneration, reduce degree of joint pain, may aid individuals postpone or prevent joint problems [69]	Little to none
Chondroitin	1200 mg per day in split doses	Osteoarthritis pain reduction	Slow cartilage degeneration, reduce degree of joint pain, may aid individuals postpone or prevent joint problems [69]	Little to none

Adapted from [84].

HMB, Beta-hydroxy-beta-methylbutyrate; BCAA, Branded-chain amino acids; EPA, Eicosapentaenoic acid; DHA, Docosahexaenoic acid; EAA, Essential amino acids; UL, Upper limit

*The reader is directed to the ISSN Position Stand on research and recommendations for a complete list on nutritional ergogenic aids of vitamins [69]

**The reader is directed to the ISSN Position Stand on research and recommendation and the ISSN Position Stand on protein and exercise [69,271]

#### 3.5.1. Beta-alanine

Beta-alanine is the rate-limiting precursor to carnosine, a powerful physiological buffer that is also linked to focus, alertness, and cognitive function during a period of stress and fatigue. The majority of research involving beta-alanine has focused on its ergogenic potential [87]. Briefly and in this respect, review papers that have summarized the literature for beta-alanine generally support its efficacy as an ergogenic aid in brief, high-intensity activities that span durations of 60–300 s [87–90]. Beyond these conclusions, one study has assessed the impact of beta-alanine supplementation (6 g/day for 4 weeks) on both physical and cognitive performance among military personnel [91]. Following a battery of physically demanding tasks, 20 warfighters were subjected to shooting tasks and cognitive assessments, whereby shooting accuracy, marksmanship, and target engagement speed improved following the 4-week supplementation period, with no observed changes in cognitive function [91]. Another study by Hoffman and colleagues [92] demonstrated that 30 days of beta-alanine supplementation significantly increased muscle carnosine content as well as improved military-specific performance assessed via a 50-m casualty carry. Additionally, significant improvements were noted for cognitive performance assessed via a 2-min serial subtraction test [92], which was performed at a shooting range while continuous fire was directed at targets nearby. Moreover, this finding highlights the ability to maintain focus during stressful, anxiety-inducing conditions (i.e., close-by gunfire) following the 30 day of beta-alanine supplementation. Future research should continue to examine beta-alanine’s potential ability to improve shooting performance and decision-making in a warfighter population.

#### 3.5.2. Caffeine

Considering its consumption in coffee, tea, cocoa, soft drinks, and other foods and beverages, caffeine is arguably the most popular nutritional supplement in the world, and this popularity is also present among military personnel [93–95]. Caffeine is a central nervous system stimulant that has the potential to enhance exercise performance and reduce fatigue [96]. Key outcomes of this substance have been critically detailed in the International Society of Sports Nutrition (ISSN) position stand on caffeine [97]. Military personnel have reported an average daily consumption of 212–285 mg/day of caffeine [95]. The Army Regulation 40-25 recommends 100 to 200 mg (100 mg per 2 h or 200 mg per 4 h) of caffeine for operational utility and during periods of sleep deprivation [67]. Operational ration components (i.e., caffeinated gum) contain 100 to 200 mg per serving and are intended to help promote optimal cognitive and physical performance among warfighters [67]. In military populations, caffeine has been reported to improve time to exhaustion during military-specific tasks [98] following sleep deprivation. Specifically, Kamimori et al. [99] demonstrated that a total daily caffeine dose of 800 mg may aid in effectively overcoming adverse effects from successive periods of extended wakefulness when optimal sleep periods are not practical.

Beyond the physical benefits, it is well documented that caffeine enhances cognitive performance and mood when appropriate doses are used [100–103]. Moreover, evidence continues to accumulate that supports caffeine’s ability to aid the warfighter during or after periods of sleep deprivation [99,104–106], particularly in terms of improving attention and vigilance, reaction time, and ability to perform complex activities, problem-solving, and reasoning [105]. Several studies have illustrated caffeine’s ability to improve marksmanship following sleep deprivation [106–108]. For example, Lieberman et al. [108] demonstrated that 200 to 300 mg caffeine doses attenuated the decrements in correct hits on a four-choice visual reaction time assessment (73% and 81%, respectively) and premature errors (98% and 88%, respectively) compared to placebo [108] during Navy SEAL candidates’ Hell Week training. This study also demonstrated that a 200 mg dose of caffeine improved traditional indicators of marksmanship: reduced distance from center of mass (23%), reduced misses (44%), increased shot group tightness (47%), and increased sighting time (23%) compared to placebo [108]. Improved sighting time was also demonstrated by Tharion et al. [106], which is a particularly relevant outcome that for this population can make the difference between life or death or correctly identifying friend versus foe.

Any individual (military operator or not) must be cognizant of the increased association of elevations in blood pressure, heart rate, heart palpitations, and general feelings of anxiousness or jitteriness that occur in some individuals after caffeine is consumed [97,109,110]. In addition, caffeine’s ability to promote wakefulness can contradict the human body and mind’s need for quality periods of rest and sleep [111,112]. This is not problematic when ingestion occurs in the morning or afternoon but can have very negative implications on both the amount and quality of sleep that an individual can expect to achieve if caffeine is consumed in the late afternoon or in the evening. Thus, careful consideration should be made by individuals who consume any dosage of caffeine within a few hours (e.g., ≤6 h) of needing to fall asleep, particularly if the dose exceeds 200 mg and/or the individual is known to exhibit sharp sensitivity to caffeine’s presence. Finally, in terms of energy drinks, data indicate that one in six warfighters reported high use (i.e., 24 oz or more/day of energy drinks consumption) and significant associations to mental health issues, aggressive behaviors, and fatigue [113]. In fact, reports suggest that military personnel consume energy drinks at a greater frequency than the civilian population [114]. Further research is warranted to assess relationships between energy drink use and these health-related variables in a longitudinal sense. Moderation is encouraged for those consuming energy drinks, and further emphasis should be given to the aforementioned considerations regarding caffeine doses [69,115].

#### 3.5.3. Creatine monohydrate

Recent reviews, including the ISSN Position Stand on creatine monohydrate, have outlined the ergogenic potential of creatine supplementation [116,117]. These reviews highlight creatine’s documented potential to increase high-intensity exercise capacity, peak power, maximal strength, repetitions completed before fatigue, and fat-free mass while undergoing physical training. While research, to date, is limited in military populations, the ergogenic outcomes observed from the use of creatine monohydrate in athletic populations are relevant to warfighters. First, Cooke et al. [118] reported that creatine supplementation might help to improve recovery and mitigate damage to muscle tissue after challenging stressful bouts of exercise. Additionally, prolonged supplementation with creatine monohydrate in competitive collegiate-level athletes has demonstrated a lower incidence of muscle-related injuries/issues, such as cramping, strains/pulls, heat illness, and total injuries [119,120]. Creatine monohydrate supplementation has also been shown to increase intracellular water and reduce heart rate, rectal temperature, and sweat rate during prolonged exercise in the heat [121]. This enhanced tolerance to performance in the heat is an important benefit to the warfighter when operating in hot environmental conditions. Recent findings also indicate that creatine monohydrate supplementation confers benefits to cognitive processes during periods of neural vulnerability, which occurs amidst stress and/or sleep deprivation [122] conditions clearly documented to occur for many military populations [123,124]. Lastly, some emerging evidence has demonstrated the ability of creatine supplementation to increase brain creatine levels by about 5–10% [122,125].

This explains some of the published outcomes supporting creatine’s ability to provide neuroprotection from traumatic brain injuries and even potential function in a prophylactic manner in terms of symptom reduction [122,126,127]. Moreover, other evidence exists to support the potential for creatine to support brain health and other associated outcomes of interest to the warfighter [124,128,129]. Most studies show that higher daily doses of creatine over a longer duration (i.e., 10–20 g/day for 2 or more weeks) may be needed to increase the brain creatine content [125,130]. Long-term high-dose creatine monohydrate supplementation (e.g., 20–25 g/day for 12 weeks and 5–10 g/day for up to 21 months) has been studied in athletic populations and have been shown to be safe and lower the incidence and severity of an injury [119,120,131,132]. Additionally, clinical populations have been studied with doses 10–30 g/day for up to 5 years [69,116,133]. The interested reader is highly encouraged to read the following reviews to further understand the potential health and performance implications of creatine monohydrate supplementation, including data that summarizes any potential safety concerns for its use [116,117,127,134].

#### 3.5.4. Key Points: Ergogenic Supplementation in Warfighters

- During sustained training and combat operations, warfighters may supplement with 200 to 300 mg/day of caffeine to enhance or combat decrements in military-specific performance.
- Available evidence suggests total daily caffeine amounts of 800 mg (200 mg doses administered with 2–3-h intervals between doses over a 24-h period) may help attenuate the negative effects of successive and chronic sleep loss (i.e., during sustained training and combat operations).
- Creatine monohydrate supplementation of 0.3 g/kg/day for 5–7 days and ~5 g/day (or 0.1 g/kg/day) thereafter is an ergogenic aid that can enhance occupational, physical, and cognitive performance, with growing evidence that it may function in a neuroprotective fashion (with daily doses of 10–20 g/day needed).

### 3.6. Preserving Whole-Body Muscle and Protein Status in Warfighters

#### 3.6.1. Essential Amino Acids (EAA)

Seminal work performed on muscle protein synthesis has highlighted the critical role that delivering an efficacious dose of EAA has on maximally stimulating muscle protein synthesis [135], while other non-protein factors also impact key components of nutrient and assimilation of skeletal muscle and other tissues [79,136]. Many studies have confirmed the benefits of EAA intake in various settings regarding skeletal muscle anabolism [137,138], lean mass [139,140], and functional outcomes [139,140] (particularly in older populations), as well as rehabilitative mitigation of muscle loss after surgery [141,142]. Further, it has long been demonstrated that free-form EAA supplementation in conjunction with exercise confers a significant anabolic effect on skeletal muscle [143] and other factors such as when the dose is ingested may also augment observed muscle protein synthesis rates [144–147]. To date, over 70 clinical trials have been conducted, indicating the benefits of EAA ingestion and/or supplementation. Further, increasing EAA ingestion has recently been demonstrated efficacious in Warfighters during periods of caloric deficit for the maintenance of body protein (see below).

Pragmatic and ergogenic reasons are evident for suggesting the inclusion of free-form EAA into the warfighter ration complement. Pragmatically, commercially available free-form EAA products are now available that are water-soluble, highly palatable, and packaged into lightweight and compact delivery formats. These formulas are suited to the warfighters who must carry their operational requirements on their person while carefully considering weight and space requirements. From an anabolic standpoint, the consolidation of research indicates that doses as small as 3–6 g are efficacious, especially when combined with exercise [148]. A maximal response with free-form EAA ingestion is derived between 10 and 15 g [135,149]. This finding is consistent with studies utilizing protein ingestion and the prevalent result that 25–30 g of protein, when combined with exercise, provides a maximal stimulation of muscle protein synthesis [150]. However, it is important to note that ingestion of free-form EAA results in greater stimulation of synthesis than an equivalent amount in high-quality protein [79].

For these reasons, warfighters are advised to consume a daily diet that delivers optimal amounts of EAA at each meal and across the entire day, irrespective of whether this occurs via intact protein sources in the form of food, isolated protein powders, or free-form amino acids. An efficacious dose of EAA is considered between 10 and 12 g [68]. Importantly, many sources of protein exist that can deliver the required amino acids [151], but an ever-present challenge for warfighters is their ability to carry large amounts of provisions and oftentimes limited access to refrigeration, while also dealing with spontaneous, chaotic changes in schedule. For these reasons and others, utilizing free-form EAA might also be a suitable alternative. Toward this aim, Church et al. [79] recently highlighted that peak EAA concentrations and EAA area under the curve are the strongest predictors of changes in the fractional synthesis rates (FSR) of muscle proteins, postprandial FSR, and whole-body protein synthesis. As such, any combination of dose, timing, source, and form in conjunction with pragmatic considerations for the warfighter that maximizes these outcomes should be most strongly considered.

For the warfighter, the EAA requirement to stimulate protein anabolism is altered in the presence of caloric deficits that often occur during periods of sustained training or combat. Largely due to the metabolic stress of exercise/high workloads and energy deficit, muscle anabolic potential is mitigated in favor of the maintenance of whole-body protein homeostasis. For example, Gwin et al. [78] demonstrated in young subjects (23 ± 5 years) that 5 days of 30% energy deficit increased the body’s need for EAA to maintain body protein balance. Under identical conditions, two EAA doses (0.10 g/kg, ~8 g/dose or 0.30 g/kg) were provided immediately after single-leg exercise. While whole-body protein homeostasis was achieved with both doses, the higher dose achieved greater rates of whole-body protein synthesis, which ultimately were two-fold greater when compared to the lower dose [78]. Follow-up work by the same research group again instituted a 30% energy deficit over 5 days [77], and this time provided a 35-g protein dose in three delivery formats: a free-form EAA + whey protein formula, whey protein alone, or a mixed macronutrient meal. A key point of this study was that muscle and whole-body protein metabolism were assessed before and after approximately 50 min of military task performance, and the authors reported that whole-body protein synthesis and net protein balance were greater with the EAA/whey combination [77]. These results demonstrate that combining optimal EAA amounts with other nutrients may further heighten the observed responses in terms of muscle protein synthesis rates and whole-body protein metabolism [152]. In addition to these outcomes, other studies have provided support in both civilian and military contexts that adding protein to the diet (which will optimize EAA delivery) in the face of energy deficits of 30–40% can mitigate lean tissue loss and optimize loss of fat tissue [153,154].

Taken together, the data indicate that efficient delivery of EAA may be beneficial to the warfighter, especially when considering the inherent operational stressors of caloric deficit, performance requirements, and the limited ability to carry nutritional intake. Adequate EAA, whether it is delivered in free-form or intact protein sources such as isolated protein powders or foods, will protect whole-body protein homeostasis during challenging physiological requirements. Thus, consideration by the warfighter should be given to effective and efficient delivery of EAA to preserve whole-body muscle and protein status.

#### 3.6.2. Key points: preserving whole-body muscle and protein status in warfighter

- A larger peripheral delivery of EAA results in greater stimulation of both whole-body and muscle protein synthesis.
- EAA ingestion formats (i.e., free-form or intact isolated proteins) provide for greater peripheral delivery of EAA and protein kinetic responses.
- Optimal delivery of EAA in the warfighter’s diet, particularly when faced with a prolonged energy deficit, can help mitigate the loss of whole-body and muscle protein and sustain higher rates of protein metabolism.

### 3.7. Additional nutritional supplement considerations

Without question, adequate nutrition and hydration are paramount when discussing nutritional considerations for tactical and service professionals. An additional area that continues to capture more interest is that of cognitive health and performance, especially as it relates to occupational performance. As outlined previously for various physical conditions, acute sleep deprivation, chronic sleep restriction, extreme environmental conditions, inadequate nutrition, and high rates of physical exertion also challenge cognitive demands and function. Beyond these challenges, the incidences of suicide [155,156] and traumatic brain injuries (TBI) [157] in military populations are two additional areas where enhancing nutrient delivery may afford positive outcomes. In this respect, interest has grown toward understanding and optimizing the potential benefits of various forms of nutritional supplementation in reference to cognitive function. While much more research is needed, two ingredients will briefly be discussed: omega-3 fatty acids and L-tyrosine for their potential to positively impact cognitive performance and health.

#### 3.7.1. Omega-3 fatty acids

Omega-3 (n-3) fatty acids are polyunsaturated fatty acids obtained through the diet or in supplemental forms and typically contain the fatty acids eicosapentaenoic acid (EPA) and docosahexaenoic acid (DHA). Researchers have referred to n-3 polyunsaturated fatty acid intake as a potential ‘nutritional armor’ for cognitive health and performance [158,159]. Briefly, eicosapentaenoic acid (EPA; 20:5 n-3) and docosahexaenoic acid (DHA; 22:6 n −3) are long-chain n-3 fatty acids endogenously synthesized from ALA. Since EPA and DHA are downstream metabolic products of ALA, they are not considered essential fatty acids. However, the conversion of ALA to EPA or DHA is inefficient and somewhat limited in humans [160]. The physiological adaptations associated with n-3 fatty acids are primarily mediated by the incorporation of EPA and DHA into tissue membrane phospholipids (e.g., skeletal, cardiac, or neural tissue). Thus, obtaining EPA and DHA from the consumption of oily fish or dietary supplements (e.g., fish oil or algal oil) is the most effective strategy to improve membrane and circulating n-3 fatty acid status.

In the brain, DHA is one of the primary structural components of the neuronal cell membrane and accounts for 10% and 97% of the total lipid and n-3 fatty acid content, respectively [161,162]. As such, preclinical models have shown that TBI reduces DHA brain content, and prophylactic DHA supplementation consistently enhances resilience to TBI, including multiple mild TBIs. Since military personnel are routinely exposed to combat and training environments that increase susceptibility to head trauma, severe and mild TBIs and the associated negative sequalae continue to be a concern for the military. While minimal human data exists, recent studies in athletes exposed to repetitive head impacts may offer some insight into the nutritional factors available to reduce the prevalence and severity of TBIs. As such, DHA supplementation (≥2 g∙d^−1^) has been shown to reduce a reliable marker of head trauma, neurofilament light chain (Nf-L), in American football players subjected to repetitive head impacts [163]. Since there are notable divergent and even competitive mechanisms of DHA and EPA, a follow-up study was conducted to determine the effect of a more standard n-3 fatty acid supplement. Heileson et al. [164] reported similar reductions in Nf-L and, thus head trauma, over the course of an American college football season with a supplement containing multiple n-3 fatty acids (2 g DHA, 0.56 g EPA, and 0.32 g DPA). In athletes, a population not unlike the Military personnel, n-3 fatty acid supplementation has also been implicated in other cognitive outcomes such as improved mood, sleep quality, episodic memory, and reaction time efficiency [165–168]. Further, evidence exists that deficiencies in n-3s, particularly DHA, may elevate deployment-induced stress that can manifest as depression and suicide [169]. Warfighters with low DHA levels are 62% more likely to have completed suicide [169]. Hallahan et al. [170] reported a 45% reduction in suicidal ideation and a 30% reduction in depression for individuals who consumed 2 g·d^−1^ of EPA+DHA for at least 12 weeks, and these data are thoroughly summarized in a review by Hibbeln and Gow [171–174]. Finally, these recommendations were also firmly supported by a military expert panel who concluded that it would be unethical not to attempt to increase the n-3 status among U.S. military personnel [175].

One of the most consistent benefits associated with n-3 fatty acid supplementation is recovery from rigorous training or exercise-induced muscle damage [173,176]. While the majority of studies used laboratory-controlled models of muscle damage, Black et al. [165] studied the effects of n-3 fatty acid supplementation during pre-season in male rugby players. Omega-3 supplementation, as a part of a protein-based drink, attenuated lower-body muscle soreness and fatigue and improved neuromuscular performance. Since recent studies have continued to report similar results such as blunted vertical jump height decrements [177], reduced delayed-onset muscle soreness [177,178], and greater range of motion [179], it is clear that n-3 fatty acids may positively influence muscle recovery following rigorous training environments.

Since Warfighters endure a high degree of physiological and psychological stress and exposure to high-risk environments with heightened susceptibility to TBIs and post TBI-associated sequelae (e.g. depression, anxiety), prophylactic n-3 fatty acid supplementation appears to be a pragmatic strategy to potentially enhance cognition protection and performance and muscular recovery. Similarly, a recent expert military panel unanimously concluded that it would be unethical to not attempt to increase the n-3 status among U.S. military personnel [175]. While the evidence for long-chain n-3 supplementation in military populations is limited (which should be a high priority area of research), there is a strong rationale for supplementation in military personnel, especially those that consume less than 2–3 servings of fatty fish per week [67].

#### 3.7.2. L-tyrosine

Tyrosine is a proteogenic amino acid that is the precursor for the synthesis of the catecholamine dopamine (DA), norepinephrine (NE), and epinephrine (E). Since tyrosine is synthesized from phenylalanine, it is not considered an essential amino acid. Importantly, however, there is evidence that the brain does not convert enough tyrosine from phenylalanine to meet its demands. This is especially true in brain-vulnerable states that arise with stress and sleep loss. In these vulnerable states, neural catecholamine activity increases and becomes more precursor sensitive [180]. Accordingly, in situations where there is increased environmental and psychological stressors or ongoing sleep loss, warfighters might find that L-tyrosine supplementation can aid with optimal cognitive functioning. Indeed, several studies have reported that various doses of L-tyrosine supplementation preserve cognitive processing under stressful conditions. For example, Mahoney et al. [181] reported that 150 mg/kg L-tyrosine supplementation prior to two 90-min cold-water immersions demonstrated improved speed and accuracy of information processing, while Shurtleff et al. indicated that a similar dosage of L-tyrosine improved matching accuracy following 30-min cold exposure [182]. Also, Coull et al. [148] demonstrated improved vigilance (9% increase) following 300 mg/kg body mass of L-tyrosine supplementation among soccer players exercising in a hot environment.

The cognitive processes affected by L-tyrosine supplementation are largely carried out through pre-frontal cortex (PFC) activity. PFC DA activity is particularly critical for cognitive processes such as working memory, decision making, task switching, attention, and general executive control functions [183]. In addition, relative to other brain areas, the PFC is more sensitive to catecholamine activity due to the PFC-unique density and activity of DA transporters (DATs) and NE transporters (NETs) [184]. It is important to note that PFC DA activity does not linearly improve cognitive processing. There is an inverted U-shaped relationship between DA activity and PFC-dependent cognitive processing such that too little *or* too much dopamine impairs performance [185]. An additional consideration in L-tyrosine supplementation for cognitive processing is that tyrosine is transported across the blood–brain barrier via a general transport mechanism for large neutral amino acids (LNAA) and thus competes with other LNAA, such as tryptophan [180]. Currently, more research that specifically seeks to identify if L-tyrosine supplantation can improve or preserve cognitive processing in warfighter environments is needed, particularly as previous research has indicated that prolonged exposure to physically demanding tasks and/or extreme conditions (sleep loss, heat, cold, altitude, etc.) can alter catecholamine production [186].

#### 3.7.3. Key points: additional nutritional supplement considerations

- Warfighters engaged in sustained training and combat likely experience decrements in cognitive performance and brain health.
- Limited but promising initial research has been completed in warfighter populations for omega-3 fatty acids and L-tyrosine. Each of these supplements currently has limited literature bases and well-controlled high-quality research investigations are strongly advised to better understand any potential benefit they may afford to warfighter’s health and readiness.
- Well-controlled investigations are needed to better understand the potential impact and translatability of the current research regarding warfighter cognition health and performance as well as mental health.

## 4. Part 2 – First responders

### 4.1. Demands of law enforcement

#### 4.1.1. Job-specific tasks and common stressors

The physiological demands of law enforcement personnel may vary considerably, ranging from those that are relatively mundane to those that are immediately life-threatening. For instance, calls for response tend to occur sporadically, which may require a law enforcement officer to quickly shift from a sedentary state to one that requires maximal physical exertion (i.e. from seated in their car or at the station to sprinting, jumping, defensive tactics, and arrest control) [2,187]. In this respect, law enforcement officers are required to pursue and apprehend suspects, enter unfamiliar scenarios (at times forcefully), engage in hand-to-hand self-defense, lift and carry heavy objects, and maneuver quickly on foot [188]. The energy demands of these activities are further exacerbated by the burden of PPE (10–40 kg) [5,189,190], which creates considerable biomechanical (i.e. postural issues, ranges of motion, and gait kinematics) and physiological constraints, subsequently also increasing the risk for musculoskeletal injury [190–193]. These challenges, when compounded over an entire career, are viewed to be primary contributors to the documented increases in injury, cardiometabolic disease, and premature death [194] in law enforcement populations.

#### 4.1.2. Physical fitness considerations and energy expenditure

During academy training, recruits are exposed to occupation-specific procedures, skills, and expected behaviors and values of a law enforcement officer [195] as well as regular physical and occupational-specific training to aid in the early development of their law enforcement career. Due to the prescriptive nature of this training period, nutritional needs, education, and shortcomings may be easier to assess. As a result, practitioners should take into consideration their physical training schedules for not only optimal physical performance but also to better prepare the recruit for real-life, on-the-job feeding scenarios. Toward this end, an emphasis on healthy eating patterns and behaviors will help promote good cardiovascular and metabolic health over an entire career. Currently, no universally accepted strength, flexibility, or aerobic capacity fitness standards exist for law enforcement, but some feel it is likely similar to the established standard for a firefighter (i.e., ~42 ml·kg^−1^·min^−1^), given the similarities in anticipated job task while dressed in full PPE and exposed to environmental stressors [196]. Further, PPE increases the metabolic cost (v O_2_; L/min) of various occupational tasks by ~6–21% depending on the weight, mode, and intensity of the work among law enforcement officers [197,198]. Specialist law enforcement personnel (such as those assigned to Special Weapons and Tactics (SWAT) officers) likely have higher aerobic fitness demands and energy expenditure due to more complex tasks and absolute load carriage (~40 kg vs. ~10 kg loads carried by nonspecialist forces, respectively) [199]. However, the specific energy needs when performing these essential job tasks is unknown, and future investigations should explore the caloric costs of performing job tasks while wearing PPE within general duty and specialist teams. Based on the daily variability in energy expenditure, the energy needs of recruits, officers, and special team members must be analyzed separately and considered when making nutritional recommendations across the occupational life span.

#### 4.1.3. Job-specific health and performance burdens

Previous research indicates that law enforcement officers are twice as likely to suffer from cardiovascular disease (CVD) compared to the general population [200]. Varvarigou et al. noted that stressful occupation-specific duties were associated with a greater risk of sudden cardiac death while on duty; however, most sudden cardiac deaths (~77%) occurred during non-routine tasks, which can all be characterized by sudden increases in cardiovascular demand from a combination of extreme physical exertion and psychological stress [201]. Similar to firefighters [202], extended sedentary periods followed by acute heavy physical exertion are occupational factors that increase the risk for sudden cardiac death and CVD in this population [201], particularly when coupled with low fitness levels [203]. Like the general population, the combination of sedentary physical activity patterns and poor dietary habits increases the risk of a law enforcement officer developing obesity [204]. Obesity rates are significantly associated with reduced overall fitness among police officers [205,206], while Williford and Scharf-Olson [207] noted that higher body fat % (BF%) was linked to poorly simulated job-specific performance. Other work has demonstrated that law enforcement personnel with higher BF% have lower aerobic capacity and reduced capacity to carry external loads compared to their leaner counterparts [208]. Moreover, Dawes et al. [209] found negative correlations between the sum of skinfolds and muscular endurance in SWAT officers, with other studies consistently showing that performance is negatively impacted by a higher BF% in addition to being at greater risk for cardiometabolic disease [210,211]. While discordance exists in the literature [212,213], it is likely that leaner law enforcement officers will perform better compared to those with excess fat mass. Thus, it is important to consider interventions (i.e., dietary and training) to 1) prevent accrual of body fat, 2) attenuate the potential negative performance effects of higher BF%, and 3) improve the overall health and fitness profile of the law enforcement officer.

### 4.2. Demands of Firefighting

#### 4.2.1. Job-specific tasks and common stressors

The job demands of firefighters can be physically arduous and environment-dependent. Structural firefighters routinely carry heavy equipment, advance unwieldy and heavy charged hose lines, climb stairs with equipment, drag/carry victims, and perform forcible entry, salvage, overhaul, and search and rescue [214], while wildland firefighters routinely build fire lines, operate a chainsaw, and hike with external loads over wildland terrain in adverse conditions [215]. Additionally, fire-resistant PPE must be worn, which adds an additional load (~26 kg for structural and ~6–20 kg for wildland firefighters) and may adversely impact both physical and occupational performance while increasing metabolic demands and cardiovascular strain [216–221]. In fact, decrements of 1.5 ± 0.43% per kg of PPE in physical ability have been noted among these personnel [221,222]. Moreover, firefighting is sporadic and unpredictable and requires performance in extreme environmental conditions [215,223]. Previous work has indicated that structural firefighting routinely yields heart rate (HR) values between 84% and 100% HR_max_, oxygen consumption values of 60% to 97% $\dot{V}O_{2max}$ in response to various fire suppression or occupational-specific tasks [224–227], and peak blood lactate levels ranging from 6 to 13 mmol/L during simulated fire ground tasks [224,228–230]. Similarly, wildland firefighters achieve HR values from 62% to 71% HR_max_ [215], with oxygen uptake levels being reported to be around 50% peak oxygen uptake (~22.2 ml·kg^−1^·min^−1^) [231] and as high as 34 ml·kg^−1^·min^−1^ during hikes [232].

It is well established that firefighters experience significant cardiac events at a rate higher than the general population [202,233,234]. Adverse cardiovascular events among firefighters most commonly occur when responding to an active structural fire [235]. It has been reported that the risk of death is 10- to 100-times greater following active fire suppression compared to non-emergency response calls [235]. The reasons for these events are certainly multifactorial, with acute and systemic changes in catecholamines, cortisol, and cytokines, such as interleukin-6 (IL-6), tumor necrosis factor-alpha (TNF-α), and interleukin-1β [236,237], which are known primary factors contributing to cardiovascular dysfunction [238]. Another key contributor is heat exposure during fire suppression training, which directly leads to fluid loss and increases in core temperature, collectively placing additional strain on the cardiovascular system [236,237,239]. Furthermore, Hunter et al. [239] reported impairments in vascular function, a 73% increase in thrombus production, and a 7% increase in platelet-monocyte binding 2 h after completion of fire suppression activities. Additionally, several other outcomes are considered to be indicative of increased cardiac stress (e.g. increases in fibrinolytic capacity, asymptomatic myocardial ischemia, and cardiac troponin I, decreases in left ventricle contractility, and stroke volume) have been shown to occur in response to varying firefighting environments [240,241]. Finally, one cannot overlook the contribution of hypokinesis, obesity, and extreme environmental conditions toward CVD among firefighters [242].

#### 4.2.2. Physical fitness considerations and energy expenditure

The current minimum standard for aerobic capacity to perform structural fire rescue tasks is 42 ml·kg^−1^·min^−1^ (with a range of 39–45 ml·kg^−1^·min^−1^) [227,230,243], and possessing this level of aerobic fitness increases the efficiency of occupational task completion and results in less fatigue during these events [230,243]. Alternatively, aerobic demands during wildland firefighting tasks can range from 10 to 34 ml·kg^−1^·min^−1^ when performing tasks such as hiking, fire-line construction, brush removal, and chain sawing [231,232,244]. Additionally, several studies have reported relationships between muscular strength, power, and anaerobic endurance with a firefighter’s ability to perform essential occupational tasks such as a victim drag, equipment hoist, ladder carry, stair climbing, and hose deployment [245–247]. Thus, high levels of both aerobic and muscular fitness have been recommended for adequate occupational performance for both structural and wildland firefighters [248].

#### 4.2.3. Job-specific health and performance burdens

As outlined in previous sections, firefighters routinely engage in high-stress activities (such as active fire suppression) that are oftentimes countered with extended periods of low physical activity. This dichotomy, combined with poor nutritional choices [249,250], ongoing and intermittent elevations in stress hormones [251–253], increased rates of obesity [254], and poor fitness levels [255], are all considered to be leading contributors to poor long-term health of a firefighter.

#### 4.2.4. Key Points: Demands of First Responders

- First responders are exposed to various physiological, psychological, and environmental stressors that increase their risk for cardiorespiratory and metabolic disease.
- First responders are routinely sleep deprived and suffer long-term consequences of sleep deprivation, which impact both their mental and physical health.
- Adequate physical fitness levels are required for optimal occupational performance and overall health, an outcome that commonly is not achieved.
- First responders need to be ready at a moment’s notice, shifting from a sedentary state to a state of high-intensity or maximal physical exertion.
- First Responders’ specific energy needs are difficult to plan against because of the unknown essential occupational tasks energy needs and the inevitable increased energy demand from PPE, load carriage, and environmental stressors (i.e. heat exposure).

### 4.3. Current health status and nutrition habits of first responders

The primary nutritional consideration for tactical athletes, particularly firefighters and law enforcement officers, is to actively take steps to first improve one’s health. Thus, it must be stressed that tactical athletes should establish and maintain a nutritious eating plan as their foundation and build toward strategies aimed at performance optimization.

#### 4.3.1. Law enforcement officers

Despite less research on this occupation, law enforcement personnel appear to exhibit similar health challenges in terms of obesity and CVD when compared to firefighters. In 2014, Can and Hendy indicated that as much as 40.5% of US law enforcement officers may be obese [256]. Currently, no consensus exists as to which approach commonly used to assess obesity (i.e., BMI, percent body fat, or waist circumference) is considered best practice for law enforcement populations. While some feel BMI may be appropriate, other data in this population suggest that BMI fails to appropriately categorize individuals as obese compared to when using BF% and waist circumference [257–259]. Additionally, CVD appears to be more prevalent among law enforcement officers, current and retirees, than among the general population (odds ratio = 1.70, 95% confidence interval = 1.03 to 2.79) [260,261]. These findings suggest that while fueling for occupational performance is important within these populations, nutritional and physical activity strategies aimed at improving general health should be prioritized as the first line of defense against the incidence of premature morbidity and mortality with these groups.

#### 4.3.2. Firefighters

For firefighters, heart attacks are the leading cause of mortality (~45% of on-duty deaths and 30% of deaths overall) [255,262,263]. Furthermore, other data suggest that firefighters have a high prevalence of CVD risk factors, with ~75% of career and volunteer firefighters being overweight or obese [255,264,265]. In addition, it has been reported that obese firefighters demonstrate higher total cholesterol, lower HDL cholesterol, higher triglycerides, higher blood pressure, and lower fitness and aerobic capacity compared to those firefighters who are of healthy weight [264]. Further, Soteriades et al. [262] found that in U.S. firefighters, CVD events in the line of duty occurred exclusively in those with underlying (non-job-related) CVD. These findings support the notion that CVD mortality in firefighters is strongly connected to poor lifestyle habits such as inadequate levels of regular physical activity and poor nutritional habits as opposed to stresses experienced during the job.

#### 4.3.3. Key Points: Current Health Status and Nutrition Habits of First Responders

- First responders demonstrate a higher prevalence of obesity, CVD, cancer, and other chronic diseases when compared to the general population.
- Nutrition practitioners are advised to focus efforts on healthy eating habits and strategies with the goal of supporting long-term health while simultaneously optimizing occupational performance.
- Like many aspects of the general population, first responders are faced with several challenges that they must overcome to improve their physical activity and improve the quality of their diet.
- Strategies such as implementing wellness policies, setting up supportive food environments, encouraging healthier food systems, and using community resources to offer evidence-based nutrition classes are inexpensive and potentially meaningful ways to improve dietary habits.

### 4.4. General energy and macronutrient guidelines for first responders

Similar to the general population, first responders should strive to consume a daily diet that provides appropriate amounts of energy, macronutrients, and micronutrients in accordance with their daily energy and nutrient needs. While little is known about the nutrient intake of first responders, guidance can be found in nutritional recommendations intended for the general population. Currently, no accurate assessment of caloric expenditure is established for on-duty first responders, and this is an area of critical need for more research. Recommended approaches to estimate total energy expenditure include using the Mifflin St. Jeor [266] or Harris-Benedict Equations [267,268] (see Table 2) with 1) the appropriate activity factor for activities of daily living and 2) the additional calories to account for those expended through physical activity (leisure and occupational) using pre-established metabolic equivalents (METs) [269] or some form of commercial heart rate and physical activity monitor that can estimate energy expenditure. However, it is important to note that these commercially available wearable devices vary considerably, and measurements of energy expenditure may be inaccurately estimated. Additional recommendations for estimating total energy expenditure would include referring to the Compendium of Physical Activity. However, the 2011 Compendium of Physical Activities lists only four specific activities for law enforcement officers and firefighters. Future research should further assess common occupational tasks to expand the Compendium of Physical Activity specific to first responder populations. The International Society of Sports Nutrition position stands outline energy and macronutrient intake guidelines for individuals engaged in general fitness programs, as well as increasing intensities of exercise [69,270], which may aid first responders depending on the intensity and volume of their exercise training. Table 3 outlines recommendations for nutritional strategies depending on the training involvement of the individual (i.e. intermediate, advanced, or endurance training) (Table 4). Additionally, the Acceptable Macronutrient Distribution Ranges (AMDRs) for the general healthy adult population are also recommendations first responders should consider [271].

**Table 2.** Basal Energy Expenditure Equations.

Mifflin St. Jeor Equations	**Men**: (10 × weight in kg) + (6.25 × height in cm) – (5 × age in years) + 5 **Women**: (10 × weight in kg) + (6.25 × height in cm) – (5 × age in years) – 161
Harris Benedict Equations	**Men**: 88.362 + (13.397 × weight in kg) + (4.799 × height in cm) – (5.677 × age in years) **Women**: 447.593 + (9.247 × weight in kg) + (3.098 × height in cm) – (4.330 × age in years)

**Table 3.** Recommended Nutritional Strategies for Individuals Involved in Intermediate Training.

*Pre-Exercise*
30–40 g carbohydrate
10–20 g protein
*During Exercise*
Water (<90 min)
Sports Drinks (>90 min, prolonged operations, and in hot/humid environments)
*After Exercise (within 30 min)*
20–40 g of protein
60–120 g of carbohydrate
*Pre-Bed Nutrient Intake*
30–40 g of carbohydrate
20–30 g of protein (primarily casein with some whey)

Adapted from [69]; Intermediate training is considered exercising 30–40 min per day, 3 times per week.

**Table 4.** Recommended nutritional strategies for athletic populations (Advanced/Endurance Training).

*General Diet*
Maintain high-carbohydrate, moderate-protein, low-fat diet that meets macronutrient intake goals
Taper training (30–50%) and carbohydrate load prior to competition
*Pre-Training/Competition Meal (3.5–4 hours before event)*
High-carbohydrate, moderate-protein, low-fat meal
*Pre-Exercise (30–60 minutes before training/competition)*
20–30 g carbohydrate
10–20 g protein
*During Exercise*
Water (<90 min)
Sports Drinks (>90 min and in hot/humid environments)
Carbohydrate/Protein Gels (intermissions)
*After Exercise (within 30 min)*
20–40 g of protein
60–120 g of carbohydrate
*Post-Exercise Meal*
High-carbohydrate, moderate-protein, low-fat meal
*Pre-Bed Nutrient Intake*
30–40 g of carbohydrate
20–30 g of protein (primarily casein with some whey)

Adapted from [69]

### 4.5. Hydration

#### 4.5.1. Considerations for first responder hydration

Optimal hydration supports vital physiological processes (i.e., hydrolysis, heat absorption, joint lubrication, waste removal, etc.), while dehydration has progressive and potentially severe implications for physical performance and cognition [272]. The Institute for Medicine recommends that 2.7 L (~11.5 cups) and 3.7 L (~15.5 cups) of total water per day are sufficient to meet the adequate intake values in 19- to 50-year-old females and males, respectively [273]. Although precise hydration guidelines have yet to be established for tactical athletes, these recommendations do appear to meet the needs of insensible water loss routes, which occur during increased respiration and perspiration when exposed to elevated temperatures. Ideally, the tactical athlete would incorporate hydration strategies to prevent dehydration beyond ~2% of bodyweight, as anything beyond this threshold has been proposed to significantly impair physical performance and cognition [274,275]. However, people, in general, are poor evaluators of their sweat loss, with recent data demonstrating that approximately one out of every two athletes (168 out of 297) underestimated their sweat loss in hot environmental conditions [276]. Although hydration guidelines are based on approximate sweat loss estimations, firefighters, and tactical athletes alike, can incorporate practical hydration strategies within their respective units from previously collected data. Cheuvront and Sawka [82] suggest a three-step approach for assessing and correcting potential hypohydration states by having athletes each morning (1) evaluate if they are thirsty, (2) monitor their urine color using the urine color chart by Armstrong et al. [277,278], and (3) weigh themselve each morning to determine if body mass is lower than normal. Based on these assessments, if an athlete has one symptom, they *may* be hypo-hydrated, if two symptoms are present, then they are *likely* hypo-hydrated, and if three symptoms are present, then they are *very likely* hypo-hydrated. Based on these assessments, practical hydration strategies outlined above can then be pursued appropriately.

Athlete recommendations for rehydration could be useful for tactical athletes as well. As pre-hydration for a firefighting event is impractical, firefighters should be recommended to first meet their daily water needs as advised by the Institute of Medicine and do so by spreading the water intake out evenly throughout the day. After physically demanding training or event response, as well as after exercise, firefighters are advised to replenish fluids at a rate of 0.4-0.8L/h of activity. In conditions of high heat/humidity or activities >2 h in duration, fluid replacement should include electrolytes [279].

#### 4.5.2. Special Considerations for Hydration among Firefighters

During any structural or active-duty fire suppression, firefighters wear PPE that provides a protective barrier from dangerously hot environmental conditions [280]. As such, PPE significantly limits the heat dissipation that occurs from sweat, resulting in increased heat accumulation and cardiovascular strain. This combination provides a continual physiological cue for sweat production that can ultimately lead to dehydration [281–283]. It has been clearly established that dehydration decreases performance [284,285], compromises thermoregulation [286–288], and impairs cardiovascular responses during exercise [289,290]. While limited data are available on firefighters’ hydration needs, it has been demonstrated that during a victim search and rescue firefighting training simulation, average skin temperature rose from 34.5 to 37.4^◦^C, average heart rate was 153 beats·min^−1^, and core temperature increased from 37.7 to 39^◦^C [291], while ambient temperatures in close proximity to the fire routinely exceed 100^o^C. Although these responses are dependent on multiple factors, this evidence clearly highlights the importance of a firefighter being aware of the challenges of their body in terms of fluid balance and thermoregulation while performing their occupational duties. Similar to recommendations for many athletes, tactical populations are advised against using thirst as an indicator of hydration status, and they are further advised to regularly assess their body mass before and after training as well as active fire suppression activities. After dehydrating physical exertion, consuming a volume of water that is equivalent to 150% of the lost body mass is advised.

#### 4.5.3. Key Points: Hydration

- Like the general public, tactical athletes are poor predictors of their hydration status and generally wait too long to begin actively hydrating.
- Dehydration is a serious threat to tactical performance as well as firefighter health. PPE exponentially decreases potential convection cooling currents as well as the evaporative potential of the skin. All told these considerations increase the rate of dehydration.
- Tactical populations should implement a three-step approach (i.e., evaluate, monitor, weigh) within their departments to better assist in maintaining euhydration.

#### 4.5.4. Diet considerations

As highlighted throughout an earlier part of this position stand, firefighters and first responders are challenged by irregular patterns of physical activity, hyperenergetic dietary patterns, and increasing rates of obesity, cardiovascular diseases, and other associated comorbidities. While both professions have the potential to operate in unique and oftentimes extremely stressful environments, very little is known regarding barriers that exist and preclude law enforcement officers and firefighters from being able to make appropriate changes to mitigate obesity development and poor fitness. From a dietary perspective, no evidence is available that warrants the recommendation of one type of dietary pattern in favor of another. Thus, all first responders who desire to maintain or improve their health should first do so by modifying their dietary habits that result in daily consumption of nutritious food that delivers an appropriate balance of energy, macronutrients, fiber, and micronutrients.

A foundational tenet of every dietary program that successfully achieves weight loss is the maintenance of a negative energy balance whereby the daily energy expended is greater than what is consumed in the diet. As advised to the general population and in position stands by Aragon et al. [292] and Donnelly et al. [293], tactical athletes who need to lose weight are advised to focus on creating a negative energy balance through a combination of energy restriction and increasing their amount of daily physical activity. While discussing the efficacy of every potential dietary strategy is beyond the scope of this position stand, approaches such as portion control, limiting the intake of energy-dense foods and ultra-processed foods, and increasing the intake of nutrient-dense foods and dietary fiber are excellent approaches upon which to focus. On the physical activity side of the energy balance equation, individuals are advised to follow ACSM guidelines for physical activity that advocate for a combination of aerobic and anaerobic activity, resulting in a minimum of 30 min of moderate-intensity exercise most days of the week [294]. While other modes, styles, and patterns of exercise can certainly be employed with high prospects of success at supporting weight loss and improving health, detailing each of these options is beyond the scope of this position stand.

As one example, the Mediterranean Diet continues to garner interest and popularity for its less restrictive approach to food choices [295] and pattern of evidence documenting its potential to reduce the risk for CVD including favorable changes in blood lipids, protection against cancer while promoting longevity [296,297], and supporting weight loss [296]. Furthermore, Yang et al. [298] previously reported that consumption of the Mediterranean Diet in a large group of Midwestern firefighters was inversely related to weight gain (odds ratio = 0.57, 95% confidence interval = 0.39–0.84), increased HDL cholesterol (*p* = 0.008), and decreased LDL cholesterol (*p* = 0.04). However, more research is needed in both firefighter and first responder populations to better understand how this eating approach can support weight loss, improvements in health, disease risk reduction, and performance optimization.

Currently, intervention approaches using firefighters and especially first responder populations are limited, with a small number of investigations having been completed using various time-restricted feeding approaches and others that have examined low-carbohydrate diets. Time-restricted feeding (TRF) has increased in popularity in recent years and collectively can be viewed as any feeding pattern that consists of distinct fasting and feeding windows [292]. In this respect, a commonly used approach involves following a 16-h fasting period and an 8-h feeding period, while other human and rodent trials involving TRF have included a range of fasting durations ranging from 10 to 21 h [299]. Previous work has illustrated the potential for TRF approaches to improve blood lipids and markers of inflammation in active, trained, and overweight populations [299–301]. Additionally, recent work by McAllister et al. [302] showed that such results achieved from TRF may not be entirely linked to caloric restriction, as individuals following a 16:8 (fasting: feeding) TRF intervention for 28 days experienced significant reductions in body fat percentage, blood pressure, and increased concentrations of HDL-cholesterol and adiponectin under both isocaloric and *ad libitum* conditions. More research is needed in different populations and for longer periods of evaluation before definitive conclusions can be drawn. In consideration of these findings, others have examined the potential impact that TRF may have on firefighter health [303]. For example, McAllister et al. [304] examined the impact of a 6-week TRF intervention among professional firefighters following a 14:10 fasting: feeding protocol. Significant reductions in markers of inflammation and oxidative stress were found, while no change was found in other markers of cardiometabolic health (lipids, glucose) or numerous inflammatory cytokines (i.e., IL-6, IL8, TNF-α). A later study by the same research group employed an 8-week TRF protocol and found reductions in IL-6 and IL-1β and reduced cortisol in response to a simulated protocol of fire ground activities (i.e., ladder carry, hose deployments, victim removal, and forced entry tasks) in firefighters [228]. In terms of performance, two studies by Moro et al. [305,306] have demonstrated the ability of TRF to improve body composition while maintaining levels of strength and endurance. In firefighters, Gonzalez et al. [307] demonstrated that 7 weeks of TRF (14:10 fasting: feeding) did not negatively impact fitness/performance variables, such as vertical jump height, one-repetition maximum for the bench press and back squat, or the number of repetitions completed for push-up and inverted row among firefighters; however, ventilatory threshold (VT) values did improve from 65.2% to 70.0% $\dot{V}O_{2Peak}$ throughout the TRF intervention. Finally, McAllister et al. [308] reported that 8 weeks of TRF (14:10 fasting: feeding) did not adversely impact time to completion during a fire simulated assessment consisting of nine occupation-specific tasks. These data suggest TRF may afford cardiometabolic health benefits to firefighters without negatively impacting physical performance; however, more work is needed to confirm these results.

While initially advocated for clinical outcomes (e.g., drug-resistant epilepsy patients) and later for reducing BF%, low-carbohydrate diets have garnered interest among tactical athletes, such as firefighters, as a means of reducing body fat and improving metabolic health and performance. In a 2015 survey, firefighters reported that the Paleo (~9%) and low-carbohydrate diets (~8%) were more frequently practiced than low-fat (~4%) or the Mediterranean Diet (1%), while diets that emphasized fruits and vegetables, as well as seafood and lean meats (i.e., Mediterranean Diet and Paleo), were viewed more favorably than diets that eliminated whole food groups [295]. To date, only one study has implemented a carbohydrate-restricted diet (CRD) in a firefighter cohort [309]. Briefly, after a 1-hour CRD nutrition counseling session, 15 firefighters followed a 28-day CRD protocol and examined markers of cardiometabolic health, CVD risk, and performance. Improvements included a reduction in fat mass (~2.4 kg; specifically, around the abdominal section), decreases in systolic (6.0 mm Hg) and diastolic (5.0 mm Hg) blood pressure, increased rates of fat oxidation (*p* < 0.05) during exercise, and several cardiometabolic markers of health (i.e. malondialdehyde, AOPP [advanced oxidation protein products], HDL cholesterol, and triglycerides). Moreover, occupational performance was also improved through observed decreases in their 2.41-km run time (~3%) and an increase in their maximal pull-up repetitions (~15%) in 2 min. Due to the small sample size and limited duration, more research is needed to further explore the potential impact a low-carbohydrate diet may have on the health and performance of first responders. In addition, a notable observation by Waldman et al. [310] highlighted the previous findings by Yang et al. [295] that indicated firefighter populations prefer a diet higher in animal proteins, which may increase feelings of satiety and naturally decrease overall caloric intake. Therefore, it is likely not the decrease in carbohydrates but rather the increase in protein consumption that makes the adoption of a CRD an effective nutrition intervention for fire communities. This observation aligns with well-established guidelines for optimizing body composition, where an emphasis is placed on calorie reduction while simultaneously increasing protein ingestion [311]. So, as seen with other parts of the general population, the increased intake of protein in place of carbohydrates during CRD protocols may aid in reducing overall caloric intake in first responder populations. Finally, low-carbohydrate, ketogenic approaches have also increased in popularity. While these diets do have some data to demonstrate their potential to reduce CVD risk and fat mass [312,313], more research is needed, especially in first responders and other such populations, before broader recommendations can be made.

In summary, several dietary approaches can be employed to help first responder populations improve their health, lose fat, and improve performance. While nearly all available studies have examined the impact of dietary interventions among firefighters, it is highly likely these interventions will offer similar benefits to law enforcement personnel. As it stands, more research is needed, particularly randomized, controlled research studies, that seek to examine the impact of any potential modulator toward health and performance are strongly encouraged. Results from these types of investigations will best inform researchers and practitioners on the potential efficacy of various exercise and diet combinations to improve the health and performance of all first responders.

#### 4.5.5. Key points: diet considerations

- First responder populations are challenged by irregular and often low physical activity patterns and poor dietary choices that commonly deliver an excess of energy, dietary fat, and cholesterol.
- Several dietary approaches exist that can aid in weight loss and improve cardiovascular health, with all efficacious approaches being grounded upon achieving a reduction in energy intake.
- The Mediterranean Diet is a preferred diet by firefighters and has been shown to effectively aid in the prevention of CVD, numerous types of cancer, and obesity.
- Initial research in TRF suggests it may help improve markers of cardiometabolic health and CVD risk in firefighters by reducing inflammation and stress markers without compromising physical performance.
- Moderately restricting carbohydrates and increasing the proportion of protein in the diet may facilitate improvements in body composition and provide sufficient satiation in tactical athletes.

### 4.6. Dietary supplementation for first responders

Dietary supplement use in the general population is widespread, with estimates suggesting that as much as 50% of US adults regularly use some form of dietary supplements [314]. Currently, the rate or pattern of usage of dietary supplements by first responder populations is unknown, and, to date, limited studies have been conducted using first responder populations. Several ingredients exist that could potentially afford some benefit to first responders, and these are outlined in Table 1. It is important to highlight that first responder populations should prioritize lifestyle changes that will help reduce their risk for obesity, cardiovascular disease, and other associated comorbidities. Dietary supplements may be a viable strategy to help first responders achieve these goals; however, very little research has been done in the area of dietary supplements in first responder populations. Subsequently, sufficient evidence to support the use of many dietary supplements within this population cannot be substantiated at this point. Certainly, there seems to be sufficient amount of literature to support the safety and efficacy of certain supplements, such as caffeine, creatine, protein, and essential amino acids, among first responder populations when used in the recommended amounts. The interested reader is encouraged to review the position stands on caffeine [315], creatine [116], and protein [68] to better understand how these supplements may be incorporated into a first responder’s regimen. Additionally, many of the recommendations put forth in the military section of this document (Part I) offer relevant insight for those working with first responder populations. Finally, while these documents are comprehensive of the current literature for these ingredients, more research is needed to better understand how the unique demands of first responder environments interact with supplementation of caffeine, creatine, and protein. For example, caffeine is well-established for its ability to improve attention, vigilance, and ergogenic properties, but more research is needed in first responder populations to better understand how caffeine’s effects interact with the (sometimes) poor physical condition, poor cardiovascular health, and extreme occupational environments experienced by firefighters. While the safety profile of caffeine is well established, future work should clearly determine what dosages or daily intakes of caffeine are suitable for these specific populations. In this respect, previous work has highlighted that caffeine dosages of 6 mg/kg may elicit a higher coagulation response during firefighting drills [316], while other studies reported that high caffeine intakes may increase core body temperature of firefighters when performance activities took place in full PPE [317]. As a result, more research is warranted to assess how caffeine intake from supplementation interacts with firefighter cardiovascular risk profiles, strenuous firefighting activities with live fire drills, and analysis of hemostatic variables following engagement in firefighting activities. Moreover, creatine monohydrate supplementation in athletes during hot and humid exercise conditions has been shown to increase body water and reduce heart rate, rectal temperature, and sweat rate. The impact of these changes on firefighters during active fire suppression is largely unknown.

Other ingredients are available that may afford potential benefits for first responders. However, very few have published literature to inform specific recommendations for these populations. Toward this aim, isolated antioxidants such as curcumin and astaxanthin have been shown to act as antioxidative/anti-inflammatory agents [318,319] that can improve aspects of cardiometabolic health [320–323], reduce biological markers of inflammation or oxidative stress, and improve recovery from muscle damaging exercise [324–327]. The challenge, however, with both of these ingredients is that their literature bases are limited in scope overall and are largely non-existent for first responders. As a result, more research is needed before firm recommendations surrounding these ingredients can be made before generalizing these results to first responder populations. Finally, one study involving ketone supplementation has examined its potential to impact oxidative stress, performance, and cardiovascular outcomes among firefighters. In this study, [328] supplemented firefighters with 7 days of an exogenous ketone salt and found no effect on pre- or post-exercise markers of oxidative stress or antioxidant status (glutathione, oxidized glutathione, superoxide dismutase, catalase, and total antioxidant capacity) [329]. In addition, the ketone salts did appear to have a small but significant cardiovascular effect as exercising HR was lowered (~3%) in firefighters while performing steady-state exercise in their PPE. While several other areas of investigation have been completed for ketone salts for both health and ergogenic outcomes, more research is needed to better understand their impact.

#### 4.6.1. Key points: dietary supplementation for first responders

- First responders should emphasize the incorporation of regular physical activity and consumption of a nutrient-dense diet to support maintaining general health, healthy weight and body composition, and occupational performance.
- First responders likely consume nutritional supplements in a similar pattern as what is observed in the general population or recreational athletes.
- Due to the unique and extreme occupational conditions upon which first responders operate, future research should determine what supplements are being routinely consumed in this population.
- While limited research has been conducted in first responders with ingredients such as caffeine, creatine, protein, and essential amino acids, the literature base on general populations of each of these ingredients provides widespread evidence of both their safety and efficacy.
- Due to the unique and extreme environments in which some first responders operate, specific research studies using caffeine, creatine, protein, and amino acid should be completed to confirm their safety and efficacy in first responder populations.
- Other ingredients such as curcumin, astaxanthin, and ketone salts have been shown to reduce markers of oxidative stress and inflammation as well as improve recovery from exercise or enhance physical performance; however, benefits in first responder populations have yet to be shown.
- Future studies should assess the efficacy and safety of any dietary supplement that is purported to aid in the improvement in health, performance, or recovery first responder populations.

## 5. Conclusion

Tactical athletes, including military personnel and first responders, work in unique occupational environments that challenge them both physically and mentally. As such, maintaining and optimizing health and performance are critical issues for all tactical athletes. As outlined throughout, appropriate fueling and hydration are critical for tactical athlete readiness and to improve health status, reduce the risk of injury and disease, and improve performance. To date, limited research has been conducted among these populations to identify fueling and hydration requirements. Currently, most of the available recommendations are derived from both general and athletic populations, and further research is needed to best understand the specific health and performance needs of these populations. While performance optimization is important, focused efforts must first be made to establish a pattern of physical activity and dietary intake that reduces the risk for cardiometabolic disease and increases fitness. As more data emerge, specific guidelines surrounding various dietary patterns and dietary supplement use will be generated, and while warfighters, firefighters, and law enforcement personnel all fall under the single term ‘tactical athlete’, the focus of provisions and timing may significantly differ across occupations and designations. As this information becomes more available, practitioners working with these populations should continually consider the occupation-specific stressors, energy expenditure, physical fitness requirements/recommendations, and job-related health burdens to which these populations are exposed in order to effectively put forth recommendations and guidelines that are practical and will promote optimal health and performance.

## 6. Position of the International Society of Sports Nutrition (ISSN)

Based on a comprehensive review and critical analysis of the literature regarding tactical athlete nutrition, conducted by experts in the field and selected members of the International Society of Sports Nutrition (ISSN), the following conclusions represent the official Position of the Society:

### 6.1. Tactical athletes

1. Tactical, or occupational, athletes perform some of the most physically demanding jobs within our society. Considerations to facilitate optimal health and performance should include the provision and timing of adequate intake of calories, macronutrients, and fluid, as well as strategic supplementation practices. Optimal nutritional practices can greatly improve physical, cognitive, and occupational performance outcomes as well as offset the impact of sleep deprivation; reduce the risk of injury, obesity, and cardiometabolic disease; and reduce the potential for a fatal mistake.
2. Personal protective equipment (PPE), load carriage requirements, and certain environmental stressors increase the demands placed upon the tactical athlete and may entail increased rate of dehydration and risk of musculoskeletal injuries depending on the occupational tasks performed. These factors are important considerations for practitioners working with these populations, especially when assessing energy and nutrient needs, which may significantly vary depending on occupation and designations.
3. Whenever possible, tactical athletes should optimize their personal health and wellness by implementing nutrition and supplementation strategies, which will also enhance occupational readiness.
4. These occupations take a steep toll on the mental and physical health and wellness of these individuals; therefore, although on-the-job best nutritional practices are needed to optimize readiness, overall health and wellness of these operators is paramount.

### 6.2. Recommendations for military

1. Military personnel are faced with various tactical-specific stressors depending on their occupation and requirements for high operational tempo and sustainment operation conditions. The current body of evidence suggests several key nutritional and supplementation strategies for the warfighter to optimize readiness while reducing the risk of injury or fatal mistakes.
2. The warfighter should aim to meet the caloric demands of occupational tasks by utilizing the Military Dietary Reference Intakes (MDRIs) established and codified in the Army Regulation 40-25.
3. Caffeine anhydrous consumption of 100 to 200 mg (100 mg per 2 h or 200 mg per 4 h) is recommended for operational utility and during periods of sleep deprivation. Doses up to 200 to 300 mg have been suggested to improve reaction time and marksmanship. Available evidence indicates that total daily caffeine amounts of 800 mg (200 mg doses administered with 2- to 3-h intervals between doses over a 24-h period) may help attenuate the negative effects of successive sleep-deprived states (i.e. sustained training and combat operations).
4. Creatine monohydrate supplementation may afford the warfighter improved exercise capacity, enhanced recovery, and/or reduced muscle damage during periods of intense physical exertion, lowered the risk for skeletal muscle-related injuries and issues (i.e., strains/pulls), enhanced heat tolerance, and improved cognition. Recommended doses are 0.3 g/kg/day (about 20 g/day) for 5–7 days followed by 0.1 g/kg/day (about 3–5 g/day) thereafter. Larger individuals may need 5–10 g/day to maintain elevated creatine stores.
5. During sustained training and combat operations, the warfighter is subjected to rapid and pronounced catabolism of whole-body and skeletal muscle protein, which can be mitigated by matching energy consumption with energy expenditure. Protein intake should be a key focus, and supplementation with essential amino acids has previously been shown to help negate protein catabolism during sustained training and combat operations.
6. Cognitive performance is critical to mission success for the warfighter. Evidence exists to support supplementation and inclusion of omega-3 fatty acids, creatine monohydrate, beta-alanine, and L-tyrosine to aid cognitive performance, especially during sustained training and combat operations and periods of sleep deprivation or chronic sleep restriction.

### 6.3. Recommendations for first responders

1. First responders are exposed to various physiological, psychological, environmental, and occupational-specific stressors that can increase their risk for cardiometabolic disease and may negatively impact duty performance. Presently, specific energy needs for occupation-specific tasks are unknown, and further research is needed to elucidate and develop better nutritional practices for first responders.
2. Little is known about the energy and nutrient intake needs of first responders in relation to physical activity levels and occupational tasks. However, it is likely that these populations would benefit from the general caloric intake and macronutrient guidelines for recreational athletes, given the nature of their occupation.
3. Nutrition practitioners are advised to focus education efforts on healthy eating habits and strategies with a primary goal to support long-term health while simultaneously optimizing occupational performance.
4. Strategies such as implementing wellness policies, setting up supportive food environments, encouraging healthier food systems, and using community resources to offer evidence-based nutrition classes are inexpensive and potentially meaningful ways to improve physical activity and diet habits.
5. A prudent guideline is for first responders to follow general nutrition guidelines and consume energy and macronutrient amounts that fall in line with the Acceptable Macronutrient Distribution Ranges for the general healthy adult population.
6. Practitioners working with first responders should estimate total energy expenditure using the Mifflin St. Jeor or Harris-Benedict Equations to appropriately estimate nutritional needs. Of note to readers, this is a critical area of need for research to establish the daily energy requirements of this population.
7. Limited research exists concerning dietary interventions in first responders. Trials on time-restricted feeding and carbohydrate-restricted dieting have shown promise in reducing adverse health risks while maintaining or optimizing physical and occupational performance among firefighters. More research is needed to investigate dietary approaches in first responders.
8. The Mediterranean Diet is preferred by the fire community and is one of the several potential dietary interventions that may improve the health of first responders. More research is needed in both law enforcement and firefighter populations to better understand the potential benefits of various dietary interventions.
9. Research concerning supplementation in first responders is limited; however, dietary supplements such as caffeine, creatine, protein powders, antioxidants, and essential amino acids may afford these individuals benefits during high-stress conditions.
